# *SF*-*1* (NR5A1) expression is stimulated by the PKA pathway and is essential for the PKA-induced activation of *LIPE* expression in Y-1 cells

**DOI:** 10.1007/s11010-015-2489-9

**Published:** 2015-06-30

**Authors:** K. Kulcenty, M. Holysz, W. H. Trzeciak

**Affiliations:** Department of Cancer Immunology, Poznan University of Medical Sciences, Poznan, Poland; Department of Biochemistry and Molecular Biology, Poznan University of Medical Sciences, 6 Swiecickiego St., 60-781 Poznan, Poland

**Keywords:** SF-1, PKA, *LIPE*, HSL, Y-1 cells

## Abstract

In the adrenal cortex, corticotropin induces the expression of several genes encoding proteins involved in the synthesis and intracellular transport of steroid hormones via the protein kinase A (PKA) signalling pathway, and this process is mediated by steroidogenic factor-1 (SF-1). This study was designed to elucidate the influence of the PKA and PKC pathways on the expression of the *SF*-*1* gene in mouse adrenocortical cells, line Y-1. It has also been attempted to answer the question whether or not SF-1 plays a role in the PKA-induced expression of *LIPE* gene encoding hormone-sensitive lipase/cholesteryl esterase, which supplies cholesterol for steroid hormone synthesis. In this study, we found that stimulation of the PKA pathway caused a significant increase in *SF*-*1* expression, and that this effect was abolished by the PKA inhibitor, H89. Decreased *SF*-*1* gene transcript levels were seen with the simultaneous activation of PKA and PKC, suggesting a possible interaction between the PKA and PKC pathways. It was also observed that SF-1 increased the transcriptional activity of the *LIPE* gene by interacting with the SF-1 response element located in promoter A. Moreover, transient silencing of *SF*-*1* expression with specific siRNAs abolished PKA-stimulated transcription of the *LIPE* gene, indicating that SF-1 is an important regulator of *LIPE* expression in Y-1 cells and thus could play a role in the regulation of the cholesterol supply for adrenal steroidogenesis.

## Introduction

The synthesis and secretion of steroid hormones in the adrenal cortex is regulated by corticotropin (ACTH), secreted by the anterior pituitary. Upon binding to a specific receptor (melanocortin type 2 receptor) via the Gs protein, ACTH activates the membrane-bound adenylyl cyclase, causing an increase in the intracellular concentration of cAMP, an activator of the PKA. Increased concentration of cAMP, and thereby increased activity of the PKA, can be achieved in vitro by treatment of the cells with forskolin, which directly activates adenylyl cyclase.

In the adrenal cortex, ACTH acts via the PKA pathway to induce the expression of genes encoding proteins involved in the synthesis and intracellular transport of steroid hormones [1–3] as well as the supply of substrate, cholesterol, stored in lipid droplets. These actions are mediated by steroidogenic factor-1 (SF-1), which induces the expression of genes encoding members of the cytochrome P450 (CYP) superfamily, as well as the genes encoding ACTH receptor, and transporting proteins including steroidogenic acute regulator (StAR) [4–6].

In the adrenal cortex, hormone-sensitive lipase/cholesteryl esterase (HSL) catalyses the hydrolysis of fatty acyl esters of cholesterol and acts as a supplier of cholesterol for steroid hormone synthesis. Numerous investigations have shown that HSL is activated by the covalent phosphorylation of Ser^563^ and Ser^660^ residues in its regulatory domain. These reactions are catalysed by a cAMP-dependent PKA whose activity is increased by ACTH at a posttranslational level [7]. HSL is encoded by the *LIPE* gene (a member of the *LIP* gene family) and is located on chromosome 19q13.3. This gene is composed of nine exons plus additional six, which are transcribed in a tissue-dependent fashion by tissue-specific promoters. In the adrenal cortex, the transcription is regulated by promoter A [8, 9] and the principal regulator of *LIPE* expression is ACTH [10].

Based on our understanding of the mechanisms that regulate the synthesis of steroidogenic enzymes, it can be assumed that HSL is controlled not only by switching on and off the catalytic activity of the enzyme, which constitutes a short-term regulation, but also by the activation of *LIPE* gene expression, encoding HSL, which represents a long-term effect. Moreover, by drawing an analogy to proteins encoded by the other genes associated with steroidogenesis, it is possible that transcription factor SF-1 affects the transcriptional activity of the *LIPE* gene in response to stimulation by ACTH.

It has been reported that *SF*-*1* expression is essential for survival and that *SF*-*1*(−/−) mice normally die at E8 due to the lack of corticosteroids, unless they are rescued by the administration of synthetic hormones [11]. SF-1 regulates the expression of genes involved in differentiation of gonads, sex determination [12] and steroidogenesis. The role of *SF*-*1* in activating of steroidogenic enzyme promoters has been defined for *CYP11A1, CYP11B1, CYP11B2, CYP17*, *CYP19*, *CYP21* and *DAX*-*1* [4, 13–15]. SF-1 activates the basic expression of these genes and additionally controls the entry of cholesterol into the cells by controlling the expression of ACTH, LDL and HDL receptors, intracellular cholesterol transporters (sterol carrier protein 2, and SCP-2) [16, 17] and the StAR protein (steroidogenic acute regulatory protein), which transports cholesterol from the outer to the inner mitochondrial membrane [18]. In addition, SF-1 also participates in the regulation of the expression of genes encoding enzymes involved in the de novo synthesis of cholesterol in steroidogenic tissues [19]. It is known that ACTH regulates the expression of steroidogenic genes via the PKA signalling pathway. However, it has not been established whether ACTH regulates the expression of *SF*-*1*. Our studies in the human adrenocortical cell line H295R provided evidence that the activators of the PKA pathway induce the expression of *LIPE* via SF-1 [20]. Activators of the PKC signalling pathway, such as phorbol esters and angiotensin II, cause an increase in the expression of HDL receptor (*SR*-*B1*) and slightly increase the transcriptional activity of the gene encoding the LDL receptor [21]. It is also possible that the activators of PKC affect the expression of *SF*-*1* and the genes encoding enzymes of the steroidogenic pathway, including HSL. Although the mechanism of interaction of PKA with PKC has not been elucidated, it is known that TPA is capable of reducing the activity of the PKA pathway through activation of PKC [21].

The aim of this study was to examine whether or not *SF*-*1* expression is regulated by PKA and to clarify the putative role of SF-1in the PKA-induced expression of *LIPE*.

## Experimental procedure

### Cell culture

Mouse adrenocortical cells (line Y-1), obtained from the American Type Culture Collection (Manassas, VA, USA), were cultured in Ham’s F-12/DMEM 1:1 (v/v) containing 2.5 mM glutamine and supplemented with 10 % foetal bovine serum (FBS), and antibiotic/antimycotic (ABAM) containing 100 U/ml penicillin, 1 mg/ml streptomycin and 100 U/ml nystatin, all from Sigma-Aldrich (USA).

### Incubation of Y-1 cells with test substances

After confluence was reached, the cells were given fresh medium (as above) without FBS. After 24-h incubation, the following substances were added: 25 µM forskolin (activator of adenylyl cyclase), 20 µM H-89 (inhibitor of protein kinase A) and 10 µM tetradecanoyl phorbol acetate (TPA; activator of protein kinase C), and the incubation was conducted for 24 h. After incubation, the cells were washed with PBS and subjected to further analyses.

### Isolation of RNA, reverse transcription and amplification of cDNA

RNA was isolated according to the phenol–chloroform method [22] using TRItidy reagent (Applichem, Germany). One μg of RNA was then reverse transcribed with the use of MMLV transcriptase and random hexamers (Novazym, Poznan, Poland), and the concentrations of the *SF1* and the *LIPE* transcripts were estimated by RT-qPCR with the use of the LightCycler 1.0 System (Roche Diagnostics, Germany) and the designed primers (Table 1). The results of RT-qPCR analysis were normalized to *MRPL19* transcript (from the mitochondrial ribosomal protein L19).

**Table 1:** Oligonucleotide primers used for RT-qPCR

Primer name	Primer sequence	Amplicon length (bp)
mSF-1 F	5′-TACTGGACAGGAGGTGGA-3′	142
mSF-1 R	5′-GAACTTGAGACAGACGAAC-3′	
mLIPE F	5′-TCCAAGCAGGGCAAAGAAG-3′	109
mLIPE R	5′-GTGTCATCGTGCGTAAATCC-3′	
mMRPL19 F	5′-AAGACGAGAGAAGGTTCCTG-3′	170
mMRPL19 R	5′-TAGGGGTCGGCTGTGGTG-3′	

Primers were designed using Oligo v.6.71 software, and DNA sequences were obtained from GenBank (http://www.ncbi.nlm.nih.gov/nuccore/)

*F* forward, *R* reverse

### Estimation of *LIPE* promoter activity using dual luciferase system

Using FuGene HD reagent (Roche Diagnostics, Germany), cells were transfected with the expression vector pCMV-SF1 containing the *SF*-*1* gene and co-transfected with two other constructs: the reporter vector pGL3 harbouring *Firefly* luciferase gene under the control of −343, or −2150 bp fragment of *LIPE* promoter A, and the pRL-TK vector, containing *Renilla* luciferase gene and used to correct for transfection efficiency. After transfection, the cells were incubated for 24 h, harvested and lysed, and the luciferase activity was determined using the Dual Luciferase System (Promega, USA) and a 20/20n Luminometer (Turner Biosystems, USA).

### Preparation of the nuclear extract and electrophoretic mobility shift assay (EMSA)

Nuclear extract was prepared by lysing Y-1 cells in low-salt buffer [100 mM HEPES pH 7.9, 15 mM MgCl_2_, 100 mM KCl, 0.1 M dithiothreitol (DTT)] and the protease inhibitor (PMSF). After separation of the cytosol by centrifugation, the nuclear fraction was extracted with high-salt buffer (20 mM HEPES pH 7.9, 1.5 mM MgCl_2_, 0.42 M NaCl, 0.2 mM EDTA, 20 % glycerol, 0.1 M DTT) and PMSF, mixed and centrifuged at 13,000×*g* for 5 min and the supernatant was used for the assay.

The double-stranded oligonucleotide:

5′-GCCGC**CAAGGTC**TCAGG**CAAGGTCA**GGGAC-3′, covering the SF-1 binding sites (underlined) within the *LIPE* promoter A, was labelled with Cy5. The binding reaction contained 10 μg of protein in 5× binding buffer (60 mM HEPES, 20 mM Tris–HCl pH 8.0, 300 mM KCl, 5 mM EDTA, 5 mM EGTA, 60 % glycerol, 1 μg poly(dC); 1 % BSA and 25 mM DTT). The reaction mixture was incubated for 20 min at 4 °C and, after adding the labelled probe (1 pmol/µl), for another 30 min under the same conditions. To verify the specificity of oligonucleotide binding to SF-1, the control reactions contained a 1:1 v/v mixture of labelled and unlabelled probes. Protein–DNA complexes were subjected to electrophoresis on non-denaturing 4 % polyacrylamide gel for 2.5 h at 75 V, and the labelled bands were visualized in a laser scanner FLA-5100 (FUJIFILM, Japan). Immediately after electrophoresis the gel was subjected to Western blot analysis using antibody directed against SF-1, followed by a HRP-conjugated secondary antibody (Santa Cruz Biotechnology, USA) and determination of peroxidase activity.

### *SF*-*1* silencing and determination of *LIPE* expression

Silencing of *SF*-*1* expression was achieved by 24-h incubation of the cells transfected with a mixture of three siRNAs (Santa Cruz Biotechnology, USA) complementary to the *SF*-*1* transcript. The effectiveness of silencing on the *LIPE* transcript level was determined by RT-qPCR, while on the SF-1 protein level the determination was made via Western blotting employing anti-SF-1 antibody and horse radish peroxidase-conjugated anti-γ-globulin (Santa Cruz Biotechnology, USA) for detection.

### Statistical analysis

The results were analysed with the aid of GraphPad InStat v.3.05 (La Jolla, CA, USA) and Microsoft Excel 2007. The results are the mean ± SEM of three independent experiments. To estimate the influence of test substances on the level of transcripts, one-way ANOVA or two-way ANOVA tests were applied. Significance of the differences between individual samples was tested at the level of *P < 0.05, **P < 0.01 or ***P < 0.001.

## Results and discussion

### The PKA but not the PKC pathway regulates transcription of SF-1

**Fig. 1:**
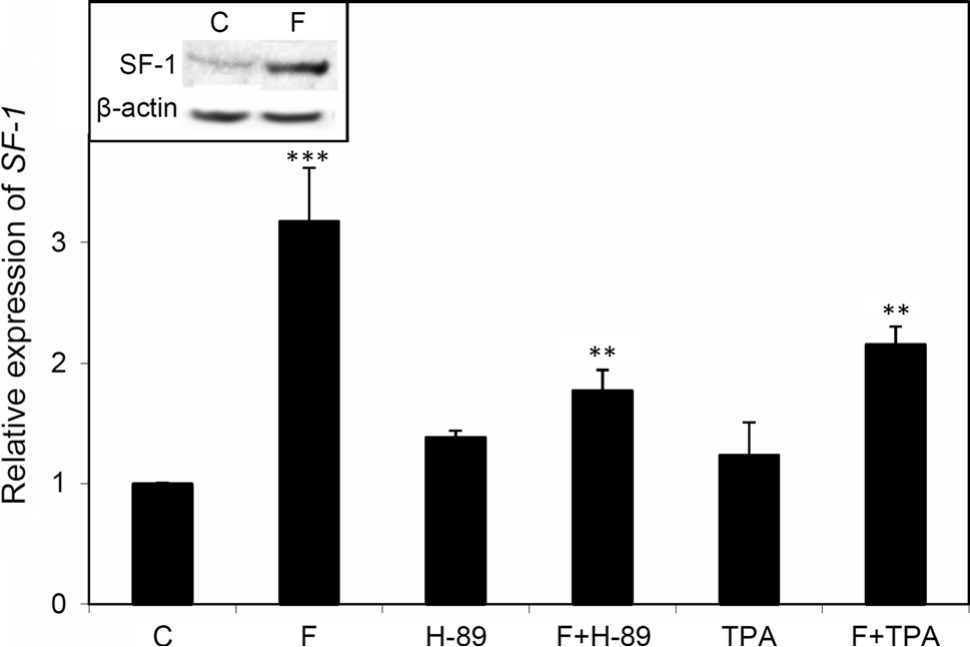
Effects of protein kinase A (PKA) and protein kinase C (PKC) stimulation on the expression of *SF*-*1* in Y-1 cells. Y-1 cells were incubated for 12 h with or without (control) forskolin (an activator of the PKA pathway), or with or without (control) tetradecanoyl phorbol acetate (TPA; an activator of PKC pathway). The incubation was followed by RNA extraction and estimation of the *SF1* transcript concentration by RT-qPCR. *C* no additions (control), *F* forskolin, *H*-*89* PKA inhibitor, *TPA* tetradecanoyl phorbol acetate. The results are the mean of three independent experiments ± SEM. The effect of forskolin on the concentration of SF-1, estimated by Western blotting, is shown in the *inset*

To investigate the effect of the PKA signalling pathway on the expression of *SF*-*1*, ACTH was replaced by forskolin, an activator of adenylate cyclase, whose effect on Y-1 cells was earlier established [23, 24]. Y-1 cells were incubated with forskolin, and after 24-h incubation, a three-fold increase in the level of *SF*-*1* transcript was observed. This was accompanied by a substantial increase in the protein product of the gene (not shown). This effect was abolished when the cells were incubated with a selective inhibitor of the PKA, H-89 (Fig. 1). Although the regulation of *SF*-*1* transcription by ACTH or cAMP in adrenocortical cells was previously investigated [25–28], the results from different studies were contradictory. In the mid-1990s, it was shown that in response to stimulation by forskolin or overexpression of the PKA catalytic subunit in Y-1 cells, the level of SF-1 protein increases, while the *SF*-*1* transcript level remains the same [25]. However, *SF*-*1* transcript levels were elevated in bovine adrenal cortex cells under identical conditions [26, 27]. The findings reported here are similar to those obtained in mouse and in bovine adrenocortical cells [28], but they are contradictory to the results reported by other laboratories investigating Y-1 cells, e.g. [29]. Such discrepancies may be due to high heterogeneity of Y-1 cell lines, especially their response to stimulation of the PKA signalling pathway [30].

It was previously established that the protein kinase C (PKC) pathway regulates the expression of some genes involved in steroidogenesis. Activators of the PKC pathway, such as phorbol esters and angiotensin II, enhance the expression of SR-B1 and slightly increase the transcriptional activity of the gene encoding LDL receptor [21]. We therefore propose that, as in the case of other genes involved in steroidogenesis, PKC might affect *SF*-*1* expression. Moreover, we hypothesize that there is an interaction between the PKA and the PKC pathways. In order to answer these questions, Y-1 cells were incubated with forskolin, TPA and with the activators of both kinases. Activation of PKC did not change the level of *SF*-*1* transcript (Fig. 1) indicating that the PKC pathway had no effect on the transcriptional activity of *SF*-*1*. Interestingly, simultaneous activation of PKA and PKC resulted in a lower level of *SF*-*1* transcript, suggesting an interaction between both pathways. It is known that PKC induces the expression of the gene encoding phosphodiesterase, which hydrolyses cAMP [21]. Therefore, PKA-stimulated *SF*-*1* transcription is probably inhibited through decreasing cAMP level.

### SF-1 stimulates transcriptional activity of *LIPE* promoter A via the PKA pathway

**Fig. 2:**
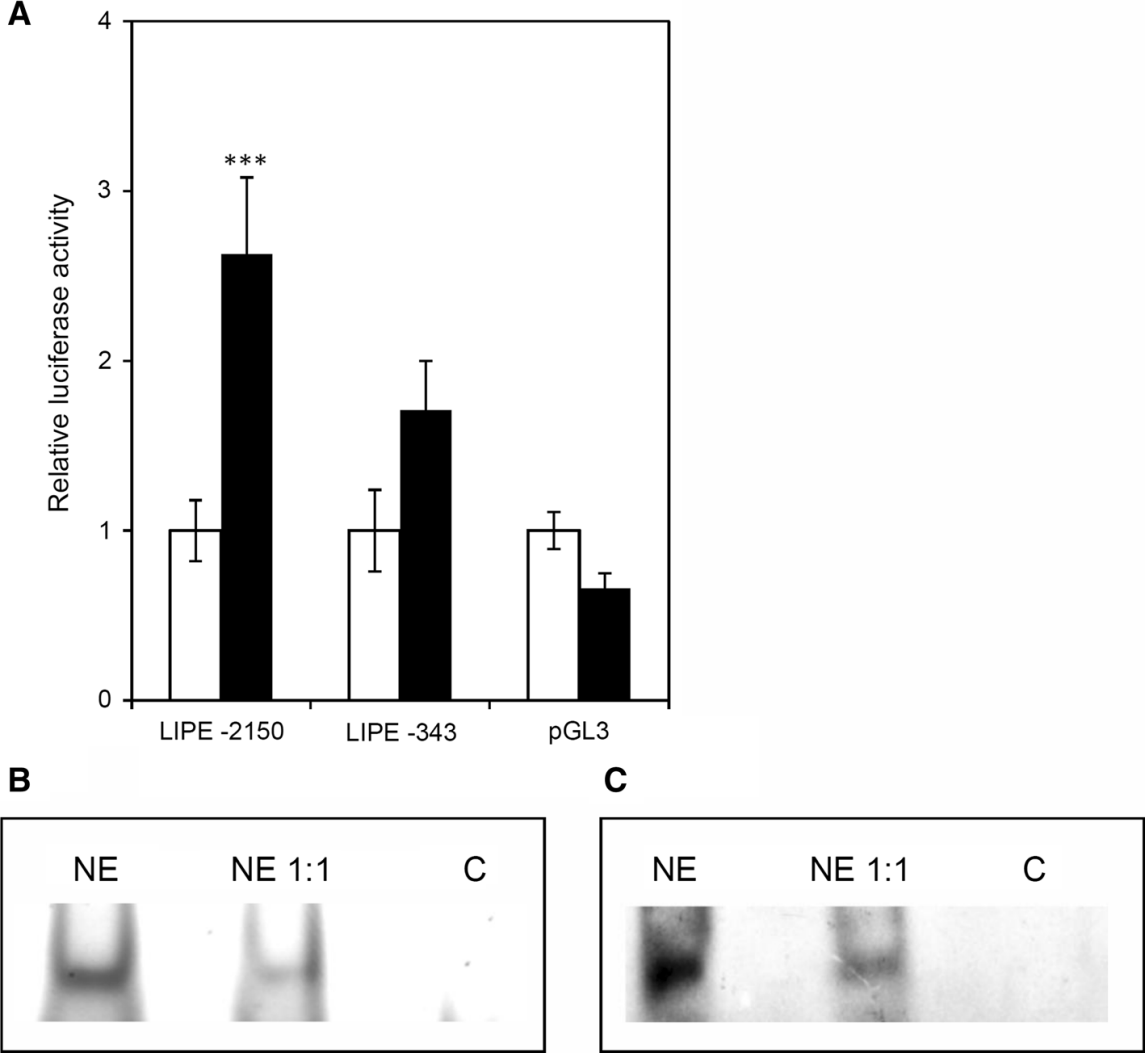
The influence of *SF*-*1* overexpression on the transcriptional activity of *LIPE* promoter (**a**) and the detection of SF-1 binding to *LIPE* promoter (**b,** **c**). Y-1 cells were transfected with *SF*-*1* expression vector and co-transfected with the pGL3 vector harbouring either −2150 or −340 fragment of *LIPE* promoter A, directing *Firefly* luciferase expression. Following 12-h incubation with (*black bars*) or without (*white bars*) the *SF*-*1* expression vector, protein was extracted and the luciferase (Luc) activity was determined by luminometry. The results were corrected for efficiency of transfection with the Luc reporter, by measuring the activity of co-transfected *Renilla* luciferase. The results are the mean of three independent experiments ± SEM. Nuclear extract (NE) from Y-1 cells was incubated with a Cy5-labelled DNA probe covering SF-1 binding sites within the *LIPE* promoter. The incubation was followed by the electrophoresis in polyacrylamide gel and visualization of the probe by fluorescence (EMSA). **c** incubation without the probe (control). The labelled protein–probe complexes were excised from the gel (**b**), and the protein was subjected to electrophoresis, blotted onto a PVDF membrane and reacted with an antibody directed against SF-1

Since SF-1 is a principal transcription factor involved in the regulation of expression of numerous steroidogenic genes [2], we presumed that it may also regulate the expression of *LIPE*. In order to investigate SF-1-dependent regulation of *LIPE* transcriptional activity, the cells were transfected with the vector containing *Firefly* luciferase gene under the control of the −343 or −2150 fragments of *LIPE* promoter A and co-transfected with the vector expressing *SF*-*1*. After 24-h incubation, the luciferase activity was determined and normalized to the transfection efficiency measured by the *Renilla* luciferase activity. *SF*-*1* overexpression resulted in an almost three-fold increase in the transcriptional activity of the −2150 fragment of *LIPE* promoter A, while there was no significant effect of SF-1 on the transcriptional activity of the −343 fragment (Fig. 2a). These results strengthen our observation, obtained from the computer analysis of the DNA sequence of *LIPE* promoter A, that within the region ranging from the −343 to −2150 bp, there are two SF-1-binding sites located within the −1400 to −1420 bp region which significantly affect *LIPE* activity.

Based on these results, we utilized EMSA to examine whether SF-1 binds directly to the *LIPE* promoter. For this purpose, we used the Cy5-labelled oligonucleotide corresponding to the SF-1 binding sites within the promoter fragment. The 5′-end-labelled oligonucleotide was incubated with 10 µg of the nuclear extract from the Y-1 cells either alone or with the mixture of 1:1 labelled and unlabelled oligonucleotide. As a negative control, the probe was also incubated without the nuclear extract. Formation of DNA–protein complexes was then monitored by electrophoresis on non-denaturing polyacrylamide gels. The formation of DNA–protein complexes was greatly reduced by the addition of unlabelled oligonucleotide of the same sequence (Fig. 2b). In order to confirm the presence of SF-1 in the DNA–protein complexes, Western blot analysis using antibody directed against SF-1 was conducted (Fig. 2c) and confirmed the direct binding of SF-1 to the response element within the *LIPE* promoter A.

**Fig. 3:**
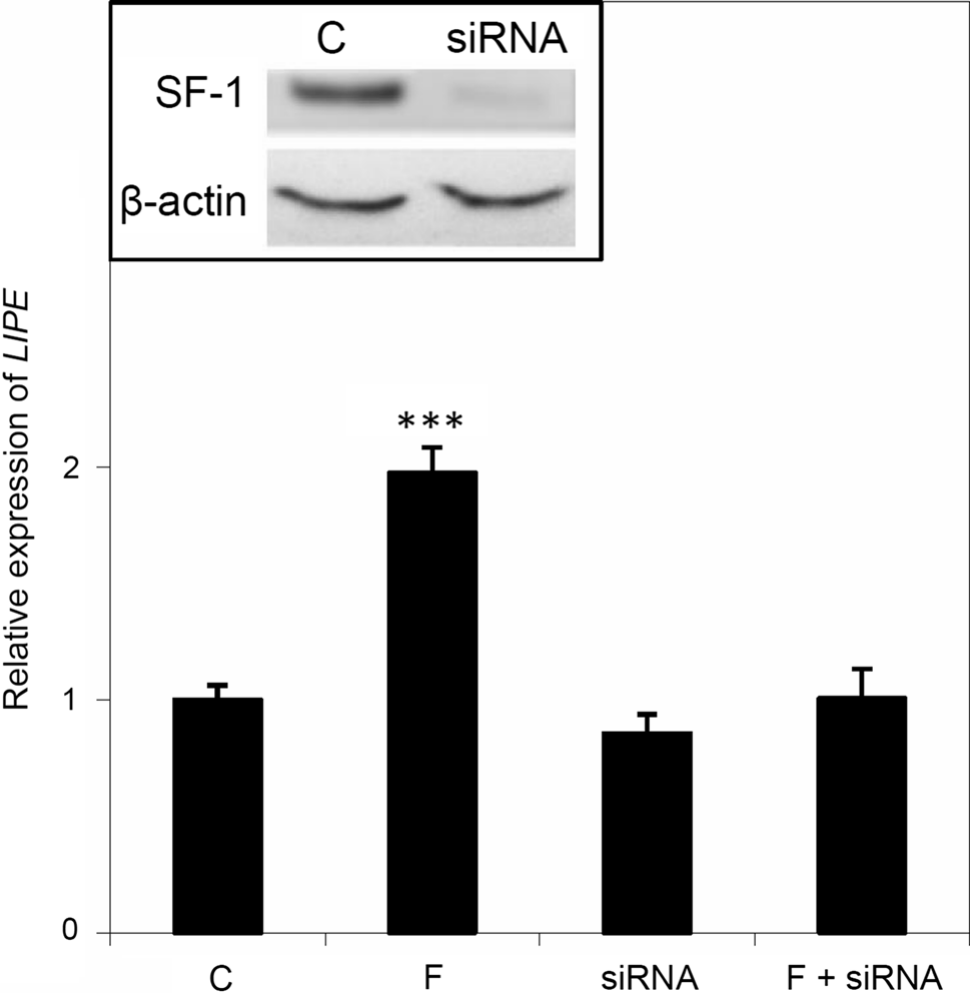
The effect of *SF*-*1* silencing on the level of *LIPE* transcript. Y-1 cells were transfected with SF-1 silencer followed by 12-h incubation: *c* without any additions, *F* with forskolin, *siRNA* with SF-1 silencer. The incubation was followed by RNA extraction and estimation of the *LIPE* transcript concentration by RT-qPCR. The results are the mean ± SEM of three independent experiments. The effect of *SF*-*1* silencing on SF-1 protein level, estimated by Western blotting, is shown in the *inset*

The results of our experiments clearly indicate that SF-1 is involved in the regulation of *LIPE* expression. However, the significance of SF-1 in PKA-dependent regulation has not been established. In order to demonstrate the significance of SF-1 in PKA-dependent *LIPE* expression, Y-1 cells were transfected with siRNA complementary to the *SF*-*1* transcript resulting in a significant decrease in the SF-1 protein 24 h after the transfection. In order to evaluate whether or not the deficiency of SF-1 affects the PKA-dependent expression of *LIPE*, control cells and the SF-1-silenced Y-1 cells were incubated for 6 h with forskolin resulting in a two-fold increase in *LIPE* expression in the control cells (Fig. 3). The silencing of SF-1 caused a significant inhibition of PKA-dependent *LIPE* expression in our experiments, suggesting that SF-1 contributes to the regulation of *LIPE* expression via the PKA pathway. We have shown that the activation of PKA is a crucial step in the stimulation of *LIPE* expression by SF-1 and that it is a major determinant in regulating cholesterol esterase/lipase expression in adrenocortical cells. However, the nature of the PKA and PKC interactions in regulating *SF*-*1* expression in Y-1 cells still remains unknown and requires further investigation.
